# A New IL6 Isoform in Chinese Soft-Shelled Turtle (*Pelodiscus sinesis*) Discovered: Its Regulation during Cold Stress and Infection

**DOI:** 10.3390/biology9050111

**Published:** 2020-05-25

**Authors:** Zuobing Zhang, Miao Tian, Ruxin Song, Xiao Xing, Yong Fan, Lan Wang, Cuijuan Niu, Roy A. Dalmo

**Affiliations:** 1 School of Life Science, Shanxi University, Taiyuan 030006, China; miaotian002@163.com (M.T.); srxsong@163.com (R.S.); xiaoxing011@163.com (X.X.); huairenyundong@163.com (Y.F.); lanwang@sxu.edu.cn (L.W.); 2 College of Life Sciences, Beijing Normal University, Beijing 100875, China; cjniu@bnu.edu.cn; 3 Research Group Aquaculture and Environment, Norwegian College of Fishery Science, Faculty of Biosciences, Fisheries and Economy, University of Tromsø—The Arctic University of Norway, N-9037 Tromsø, Norway

**Keywords:** Interleukin 6, IL6, Chinese soft-shelled turtle, acute cold stress, infection

## Abstract

The Chinese soft-shelled turtle (*Pelodiscus sinesis*) is a widely cultured commercial species in East and Southeast Asian countries. The turtles frequently suffer from acute cold stress during farming in China. Stress-induced factor such as Interleukin-6 (IL6) is a multifunctional molecule that plays important roles in innate and adaptive immune response. In the present study, we found that the turtle possessed two IL6 transcripts, where one IL6 transcript contained a signal peptide sequence (*psIL6*), while the other IL6 transcript (*psIL6ns*) possessed no such signal peptide gene. To test any differential expression of the two isoforms during temperature and microbial stress, turtles were adapted to optimal environmental water temperature (25 °C), stressed by acute cooling for 24 h, followed with the challenge of *Aeromonas hydrophila* (1.8 × 10^8^ CFU) or *Staphylococcus aureus* (5.8 × 10^8^ CFU). Gene characterization revealed that *psIL6ns*, a splicer without codons encoding a signal peptide and identical to the one predicted from genomic sequence, and *psIL6*, a splicer with codons encoding a signal peptide, were both present. Inducible expression was documented in primary spleen cells stimulated with ConA and poly I: C. The splenic and intestinal expression of *psIL6ns* and *psIL6* was increased in response to temperature stress and bacterial infection.

## 1. Introduction

Interleukin-6 (IL6) is a pleiotropic cytokine that functions not only in immune responses, but also in a range of other biological/physiological processes, such as in acute-phase reactions and hematopoiesis [1,2]. In mammals, the biological activities of IL6 are mediated by the IL6R-gp130 receptor complex in the plasma membrane. IL-6R/gp130 complex contains two type I transmembrane proteins, the ligand-binding α-subunit receptor IL6R and the signal transducing β-subunit, gp130 [1]. The signal transduction pathway JAK/STAT, ERK, and PI3K are in the downstream of IL6 activation [1].

IL6s have been documented in many species spanning several vertebrate taxon. In mammals, the structures and functions of IL6s in human and mice have been well characterized at the genomic, transcriptomic, and protein level. *il6* genes have also been described in avian species [3,4]. *il6* homologs have been found in a number of fish species as well, including pufferfish (*Fugu rubripes*) [5], flounder (*Paralichthys olivaceus*) [6], gilthead seabream (*Sparus aurata*) [7], rainbow trout (*Oncorhynchus mykiss*) [8], zebrafish (*Danio rerio*) [9], orange-spotted grouper (*Epinephelus coioides*) [10], large yellow croaker (*Larimichthys crocea*) [11], and blunt snout bream (*Megalobrama amblycephala*) [12]. The IL6 homologs in fish and birds were found to have similar functions as their counterparts in human and mice [10,13]. However, any scholarly reports on reptilian IL6 is not made yet.

The Chinese soft-shelled turtle (*Pelodiscus sinesis*) is an economically important aquaculture species in China and its draft genome is available [14,15]. In our previous study, it has been reported that the deduced IL6 of the Chinese soft-shelled turtle, retrieved from genomic sequence data, contains no signal peptide [16]. This is in contrast with common knowledge that as a secretory protein, IL6 should possess a signal peptide. Consequently, we did extensive data mining for IL6s of turtles, which were deposited in GenBank, namely the western painted turtle (*Chrysemys picta bellii*, Acc. no. XP_008169102), green sea turtle (*Chelonia mydas*, Acc. no. XP_007066238), and Goodes thornscrub tortoise (*Gopherus evgoodei*, Acc. no. XP_030405239). Interestingly, it was found that sequence of any signal peptide was not present/not submitted for all their IL6s, which means that they may not play similar roles to their mammalian, avian, and teleost IL6 counterparts. In our previous work, we found that the Chinese soft-shelled turtle *il6* was induced upon *Aeromonas hydrophila* challenge [16]. Moreover, the Chinese soft-shelled turtle *gp130*, which is a component of IL6 receptor complex, has been characterized in another study [15], and downstream molecules JAKs, STATs, and PI3Ks have also been deposited in GenBank. The Chinese soft-shelled turtle frequently suffers from acute cold stress in China. This temperature-induced stress causes heavy economical loss for farmers due to death [16]. IL6 leads to energy mobilization, and thus elevated body temperature [17], and is considered as an anti-inflammatory mediator/cytokine when released by muscle cells and pro-inflammatory when produced by e.g., macrophages/monocytes [18]. The role cold-induced stress has on IL6 expression during bacterial infection adds additional interest to investigate IL6 in the turtle, as infections may be detrimental to the turtle’s welfare—especially during cold periods. We previously described that the acute cold-stressed Chinese soft-shelled turtle induced *il6* expression in response to bacterial infection [16]. Unfortunately, in this study, only one sampling time point was sampled. This is too scarce to fully depict the response curve of *il6*. Therefore, we extended the present study by including several sampling time points to determine the dynamics of *psIL6ns* and *psIL6* expression in cold-stressed turtles experimentally infected by bacteria—in an attempt to obtain a better comprehensive understanding of the response curve. The research hypothesis is that the two IL6 variants display different expression patterns during temperature stress and during bacterial infection.

## 2. Materials and Methods

### 2.1. Animal Holding and Bacterial Infection

Eight healthy Chinese soft-shelled turtles (99.21 ± 21.24 *g*) were purchased from a farm in Yutian, Hebei Province, China. The animals were without any clinical signs of infection or disease. The animal experiment was performed according to the Guideline on the Humane Treatment of Laboratory Animals (http://www.most.gov.cn/fggw/zfwj/zfwj2006/200609/t20060930_54389.htm).

The turtles were fed commercial standard diets (HebeiHaitaitech. Ltd., Shijiazhuang, China) daily. After acclimation at 25 ± 1 °C for two weeks, the liver, spleen, kidney, lung, stomach, and intestines (distal ileum and large intestine) were sampled for basal expression analysis of target genes. The intestine was differentiated according to the previous report [14].

Two hundred and forty turtles (116.56 ± 25.30g) were purchased from the same farm and acclimated as described above. After acclimation, turtles were randomly divided into six groups. Three groups were maintained at 25 ± 1 °C and injected intraperitoneally with PBS, *Aeromonas hydrophila* (1.8 × 10^8^ CFU per animal) or *Staphylococcus aureus* (5.8 × 10^8^ CFU per animal), respectively. With a cooling-water machine, the water temperature for the other three groups was lowered to 15 °C and kept at this temperature for 24 h. Animals in these cold-stressed turtles were infected as described above and kept at 15 °C for 7 d. The immune organs, spleen and intestine (distal ileum), were sampled from 8 replicate individuals at 6, 12, 24, 72 h and 7 d. In another experimental, for oral infection, two juvenile turtles after receiving two-week acclimation were administrated with 1 × PBS and *Aeromonas hydrophila* (1.0 × 10^9^ CFU), respectively. After 24 h at 25 ± 1 °C following bacterial administration, the spleen, distal ileum, large intestine, and brain were sampled for RNA extraction.

### 2.2. Total RNA Isolation, cDNA Synthesis, Real-Time RT-PCR, and Semi-Quantitative RT-PCR

Total RNA from the collected tissues was isolated according to a method previously described [19]. The protocol described by Zhu et al. [20] and PrimeScript™ RT reagent kit with gDNA Eraser (TAKARA, Cat. No. RR047A) was used for cDNA synthesis. For each sample in the oral infection experiment, mock control (RNA) was set in parallel with cDNA, and the reaction system was the same as that of cDNA synthesis, except that reverse transcriptase (PrimeScript RT Enzyme Mix I, Takara, Dalian, China) was replaced by ddH_2_O.

Real-time PCR was performed in duplicates with an ABI PRISM 7500 Sequence Detection System (Applied Biosystems, Singapore). The reaction procedure followed a previous method [16]. The reagent was 2 × SYBR Green PCR Master Mix (Applied Biosystems, Cat. No.4367659). As illustrated in Supplementary Figure S5, the reverse primer of *psIL6ns* is specifically located in the additional 125 bp insertion which is not present in *psIL6* transcript. The forward primer of *psIL6* spans the junctions of exon1 and exon 2 with 3 nucleotides in exon 2. The amplicons of *psIL6* and *psIL6ns* were verified by sequencing.

The *il6* primers for semi-quantitative RT-PCR could potentially amplify both *psIL6* and *psIL6ns*. The size of *psIL6* and *psIL6ns* amplicons was 222 and 347 bp, respectively. H_2_O was used as the template to exclude any environmental contamination, and mock control was used as the template to rule out the possibility of any genomic DNA contamination. The obtained gel bands were cloned and sent for Sanger sequencing. The primers are listed in Supplementary Table S1, and *elongation factor 1 α* (*ef1α*) was used as the reference gene in both qRT-PCR and semi-quantitative RT-PCR [16].

### 2.3. Cloning of Turtle IL6 cDNA

In GenBank, a nucleotide sequence (Acc. No. XM_006138351.2), predicted by automated computational analysis, was denoted as *Pelodiscus sinensis IL6*. Based on this sequence, primers for RACEs were designed (Supplementary Table S1). 3′-and 5′-RACE ready cDNA library were constructed with a SMARTer RACE cDNA amplification kit (Takara, Cat No. 634923) by using total RNA mixed from the tissues mentioned above. For *IL6* cDNA verification, nested PCRs were carried out, and the amplicons covered the complete coding sequence. The T-vector pMD-19T simple (Takara, Cat No. 3721) was used for constructing sequencing plasmids. Positive clones were selected for sequencing.

### 2.4. Bioinformatic Analysis of the Target Sequences

The obtained sequences were bioinformatically analyzed as previous [20]. In brief, protein sequence was deduced by the online program TRANSLATE in ExPASy (https://web.expasy.org/translate/). Protein domain analysis was performed using the Simple Modular Architecture Research Tool (SMART) [21], and the existence of signal peptide was investigated using the SignalP v4.1 Server [22]. Secondary structure was further predicted with the online software SOPMA [23]. Multiple sequence alignment of IL6 was carried out by using Multiple Sequence Alignment (MUSCLE) software [24] by choosing selected animals known to represent evolutionarily important branches (see Supplementary Table S4 for accession nos.). Phylogenetic relationship was analyzed using the neighbor-joining method by MEGA X [25] with Johns–Taylor–Thornton model and bootstrap test for 1000 replicates, and Gamma value was set as 1.676610112 which was obtained in the model selection test. Similarity and identity at amino acid level were run with BLASTP. The introns and exons of *IL6* in selected animals were manually identified in ENSEMBL [26]. Gene synteny analysis was also carried out in ENSEMBL.

### 2.5. Primary Cells Stimulation

The adult turtle spleen was sampled and washed in pre-cooled 1 × PBS containing antibiotics (500 U·mL^−1^ penicillin and 500 U·mL^−1^ streptomycin) trice. With a plunger from a 2 mL disposable syringe, the spleen was passed through a 150 μm pore size cell strainer in pre-cooled M199 medium (Hyclone, Cat. No. SH30253.01) without serum. The filtered spleen cells were centrifuged and sedimented at 1000 rpm at 4 °C for 5 min. After being washed with pre-cooled M199 medium at 1000 rpm at 4 °C for 5 min, the pellets were resuspended with M199 medium with 10% FBS (Every Green, Cat. No.11011-8615). The cell numbers were adjusted to 1 × 10^6^ per mL and 1 mL was added into each well (in total three 24-well cell culture plates) (Nunc, Cat. No. 142475). After 24 h incubation at 26 °C, the culture medium was replaced with 1 mL fresh M199 medium with 10% FBS, 500 U·mL^−1^ penicillin, and 500 U·mL^−1^ streptomycin. 1× PBS, Con A (25 µg per mL, Sigma, Cat. No. L7647), and poly I: C (5 µg per mL, Sigma, Cat. No. P1530G) were subsequently added into 6 wells at each plate, respectively. The cells were stimulated for 3, 6, 12, and 24 h and the cells in each well were harvested and fixed using in 500 µL RNAiso Plus (Takara, Cat. No. 9109) reagent after washing with PBS. Total RNA was isolated and reversely transcribed into cDNA. Expression of target genes in the cells was determined by real-time PCR. The experiment was repeated once.

### 2.6. Statistical Analysis

Real-time PCR data were analyzed using SPSS v19.0 (IBM) or GraphPad Prims 6 (GraphPad Software, San Diego, CA, USA). When a parametric method was found to be applicable, the homogeneity of variance of these data was first tested, one-way ANOVA analysis or two-way ANOVA analysis was carried out followed by Tukey’s method or Bonferroni’s multiple comparison test in the multiple comparison. When a non-parametric method was found applicable, Kruskal–Wallis analysis was first used and when there was a significance (*p* < 0.05), the Mann–Whitney U test was used as a post hoc test.

## 3. Results

### 3.1. Sequence Analysis and Characterization of Turtle IL6

**Figure 1:**
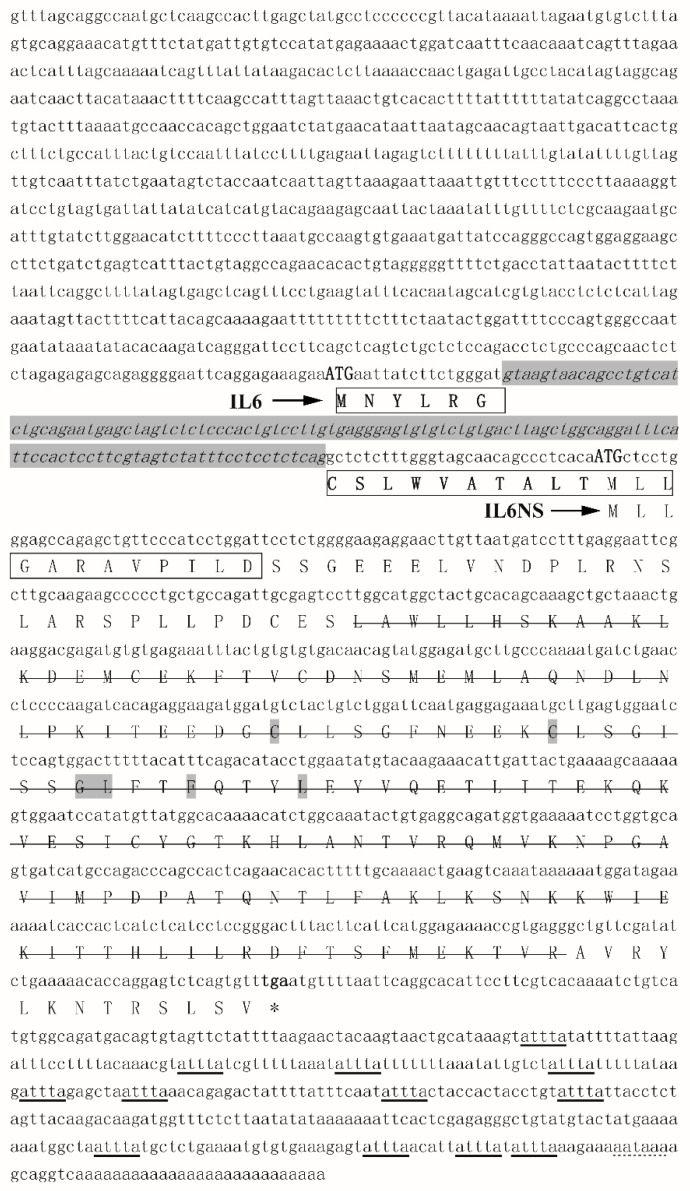
Nucleotide and deduced amino acid sequences of *psIL6* and *psIL6ns*. Additional nucleotides of *psIL6ns* are in italic and shaded. Translation starting sites for *psIL6* and *psIL6ns* are in uppercases. Signal peptide of *psIL6* is boxed. The IL6-superfamily domain has a single strikethrough. The IL6/G-CSF/MGF family signature characteristic (C-X(9)-C-X(6)-G-L-X(2)-Y/F-X(3)-L) is shaded. Instability motifs (attta) are underlined and polyadenylation signal (aataaa) is above the dashed line.

Based on the results of 3′- and 5′- RACE, as well as the sequences deposited in GenBank, Chinese soft-shelled turtle *IL6* cDNA in full length were assembled, and the amplicon covering open reading frame (ORF) was further confirmed by PCR and sequencing. Two splicers were identified (Figure 1 and Supplementary Figure S1), which were named *psIL6* (Acc. No. MK038868) and *psIL6ns* (Acc. No. MK038869). *psIL6* cDNA was found to be 2069 bp in length, with a predicted 663 bp-long ORF and putatively encoding a protein with 220 amino acids with theoretical PI of 6.83 and molecular weight of 24.8 kD. The 5′- and 3′-UTR of *psIL6* were 970 and 436 bp long, respectively. *psIL6ns* was found to be 2194 bp in length, with a deduced 615 bp-long ORF and putatively encoding protein with 204 amino acids with theoretical PI 6.33 and molecular weight 23.0 kD. The result from the alignment of the two splicers showed that there was an additional 125 bp insertion in *psIL6ns* (Figure 1 and Supplementary Figure S2). A signal peptide containing 28 amino acids was predicted in *psIL6* at the N terminal end. No signal peptide was found in *psIL6ns*. Polyadenylation signal (AATAAA) and multiple instability motifs (ATTTA) were found in 3′-UTR in both genes. The core sequences of *psIL6* and *psIL6ns* were manually identified (Figure 1). IL6/G-CSF/MGF family signature characteristic (C-X (9)-C-X (6)-G-L-X(2)-Y/F-X(3)-L) was found in both amino acid sequences.

**Figure 2:**
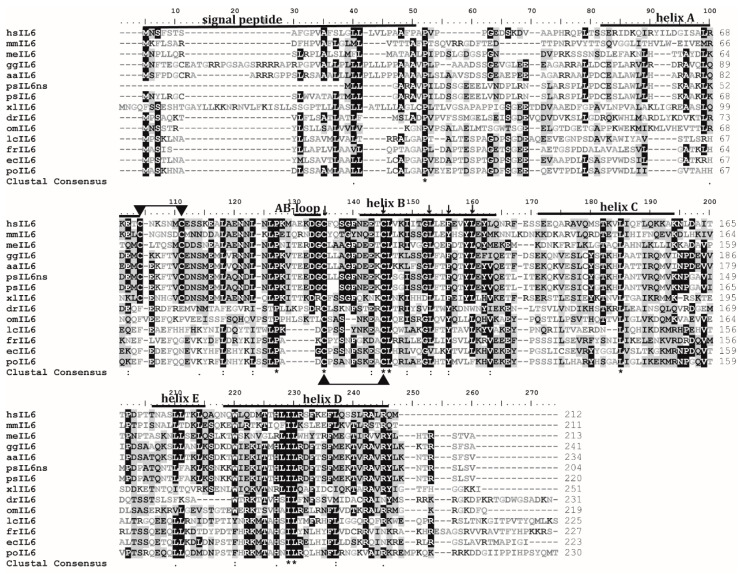
Multiple sequence alignment of the deduced IL6 in the Chinese soft-shelled turtle and other selected animals. The consensus residues are shaded. In the consensus line, asterisks (*) resemble completely identical residues in all selected species, and dots (.), and colons (:) represent similarity. MUSCLE program was used for the alignment. Accession numbers of genes are supplied in Supplementary Table S4 and the first two letters of the sequence name represent the initial letters of species’ Latin name. Signal peptide, Helix A-E, and AB loop of human IL6 are clearly denoted. Conserved cysteines are indicated with triangles and putative disulfide bonds are linked with single lines. Critical amino acid residues in the IL6/G-CSF/MGF family signature characteristics were labeled with solid arrows.

The results of the BLAST search applied with *psIL6* and *psIL6ns* nuclear sequences in GenBank suggested that they were homologous to mammalian *IL6*. Moreover, the alignment of *psIL6* and *psIL6ns* with IL6s from other selected vertebrates demonstrated that they are conserved at a certain level. IL6 in the Chinese soft-shelled turtle was more conserved among higher vertebrates such as in human, birds, and turtle than that in fish (Figure 2). The Matrix Global Alignment (Supplementary Table S3) showed that *psIL6* and *psIL6ns* possessed highest similarity (95%) and identity (90%) to the western painted turtle, secondarily highest similarity (80%) and identity (68%) to chicken (*Gallus gallus*), and lowest similarity (23%) and identity (37%) to pufferfish (*Takifugu rubripes*).

### 3.2. Gene Synteny, Genomic Structures, and Phylogenetic Relationship Analysis

The BLAST analysis of the two cDNA in the Chinese soft-shelled turtle genome suggested that they were transcribed from one gene, which was denoted as *IL6*. Gene synteny analysis (Supplementary Figure S3) in *IL6* loci demonstrated that *sp4* and *cdca7l* genes are in the left flank and the transcription orientation of the two genes are conserved in the selected animals (Chinese soft-shelled turtle, human, chicken, and pufferfish). The *tomm7* gene is in the right flank of the *IL6* gene in human, turtle, and pufferfish, while zebrafish *tomm7* gene flanks left of the *IL6* gene with inversed transcription orientation [9]. The *klhl7* gene is observed in the right flank in human, chicken, and turtle.

The genomic sequence of *IL6* ORFs in human, chicken, pufferfish, and zebrafish spans 5 exons and 4 introns (Supplementary Figure S2). *psIL6* ORF possesses the same genomic structure while *psIL6ns* ORF only has three introns. The relationship between the turtle *IL6* genomic sequence and *psIL6* or *psIL6ns* cDNA is shown in Supplementary Figure S2b, where *psIL6ns* keeps the first intron in *psIL6* during RNA splicing. This does not shift the translation frame, but the translation starts from a downstream “ATG” by prediction, which makes *psIL6ns* a part of *psIL6*.

**Figure 3:**
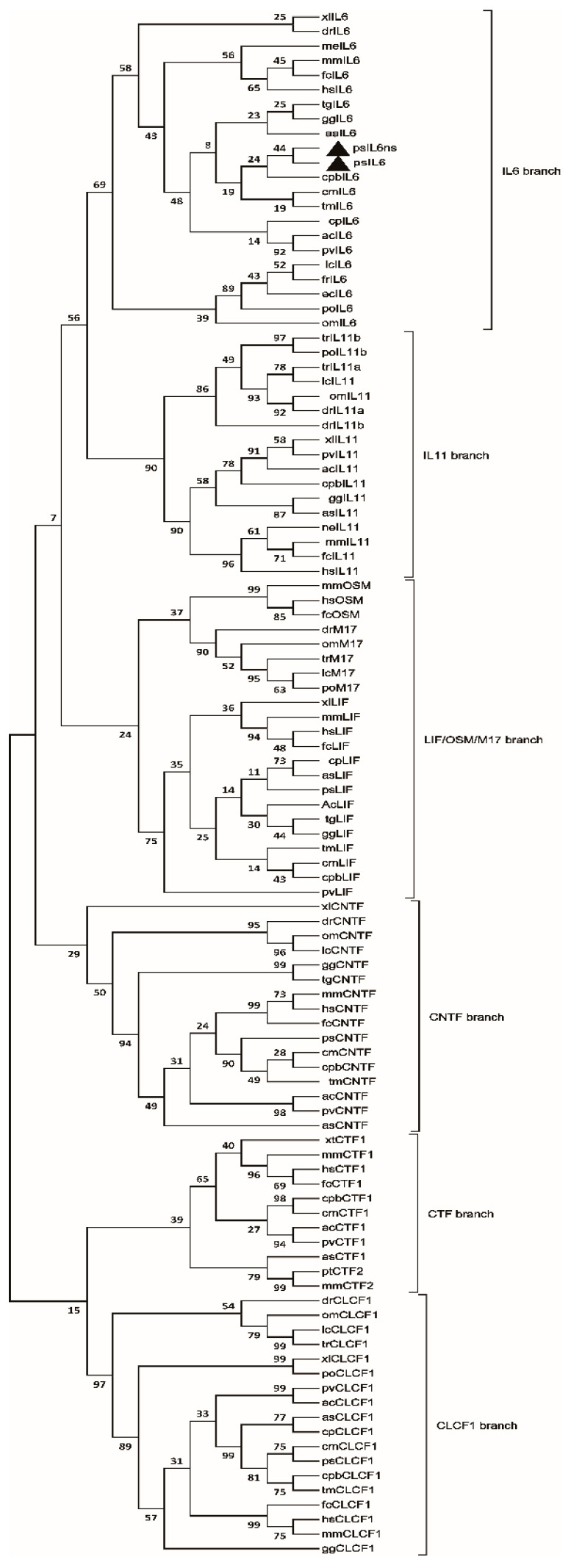
Phylogenetic tree showing the relationship between the turtle *IL6* gene and genes of *IL6* families in other selected vertebrate species. The phylogram was constructed on MUSCLE and MEGA X. The neighbor-joining method was used. Johns–Taylor–Thornton model with Gamma value of 1.676610112 and bootstrap values of 1000 replications were adopted. Accession numbers are supplied in Supplementary Figure S4. *psIL6* and *psIL6ns* are labeled with a filled square.

A phylogenetic tree (Figure 3) was constructed including members of the IL6 family in the selected vertebrates. Six branches, named IL6, LIF/OSM/M17, CNTF, CTF, CLCF1, and IL11 branch, were identified in the tree. *PsIL6* and *psIL6ns* were found to be in the IL6 branch, which is further divided into the teleost IL6 sub-branch and a non-fish vertebrate one. *PsIL6* and *psIL6ns* were placed in the latter sub-branch. Our two IL6s groups initially with the western painted turtle IL6, then they, together with the chicken IL6, group with the mammalian IL6 cluster (human and mice IL6).

### 3.3. Tissue Distribution of psIL6 and psIL6ns in Healthy Animals

**Figure 4:**
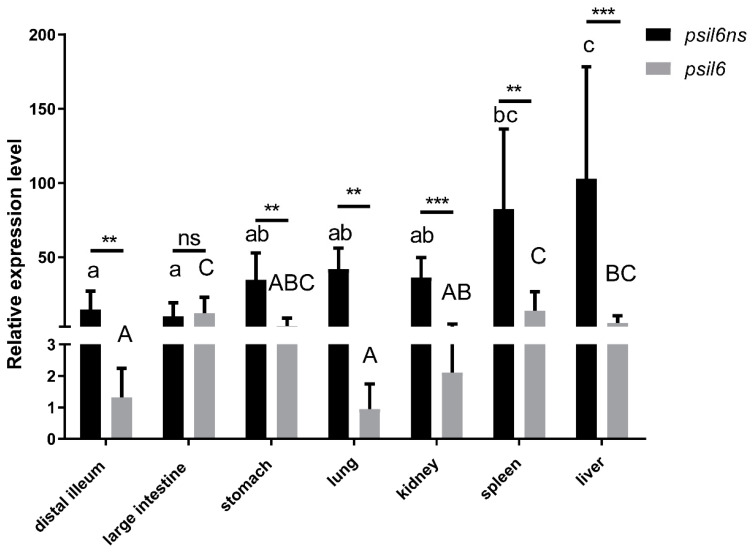
The constitutive expression of *psIL6* and *IL6ns* mRNA was determined by real-time PCR in seven tissues from eight turtles. The results were calculated in a relative expression method, and presented as mean + SD. *ef1α* was chosen as the reference gene. If there is not any same letter (uppercased vs. uppercased, lowercased vs. lowercased) in any two different groups, it represents that there is a statistical significance (*p* < 0.05); ** *p* < 0.01; *** *p* < 0.001. One-way ANOVA analysis was performed to analyze the data in different tissues and Tukey’s method was applied as a post hoc test. Unpaired T test was used to compare *psIL6* and *IL6ns* transcript levels in the same tissues.

The mRNA expression levels of *psIL6* and *psIL6ns* in selected tissues were determined by qRT-PCR analysis (Supplementary Figure S5). As shown in Figure 4, the expression of both splicers was found in the selected tissues. In general, the mRNA level of *psIL6ns* was significantly higher compared to *psIL6* (distal ileum: *p* = 0.0037; stomach: *p* = 0.004; lung: *p* = 0.004; kidney: *p* = 0.0007; spleen: *p* = 0.0059; liver: *p* = 0.0003), with one exception being the large intestine (*p* = 0.6943). Tissue-dependent expression of *psIL6ns* was found (*p* < 0.0001). The liver and spleen expressed the highest level of *psIL6ns* mRNA, whereas stomach, lung, and kidney expressed mid-levels, while intestine expressed a low level of *psIL6ns*. Significant different expression of *psIL6* (*p* = 0.001) was also observed in the different tissues. The level of *psIL6* mRNA was high in the spleen and large intestine, moderate in the liver, stomach, and kidney, and low in the distal ileum and lung.

### 3.4. Regulation of psIL6 and psIL6ns Expression in Primary Spleen Cells

**Figure 5:**
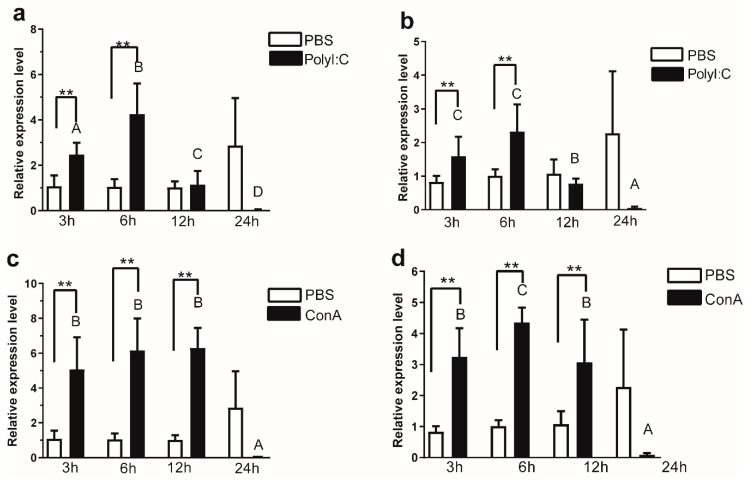
Transcript levels of *psIL6* and *psIL6ns* in primary spleen cells upon stimulation. The relative expression method was used in the calculation with *ef1α* as the reference gene. In the *X*-axis, stimulation times (h) are listed. (**a**) *psIL6* and (**b**) *psIL6ns* expression after poly I: C (5 μg mL^−1^) stimulation, (**c**) *psIL6* and (**d**) *psIL6ns* expression after ConA (25 μg mL^−1^) stimulation. The data are presented as mean + SD (*n* = 6). Different letters above the bars represent statistical significance between the time-points (*p* < 0.05); ** *p* < 0.01; *** *p* < 0.001. Two-way ANOVA analysis was carried out followed by Bonferroni’s multiple comparison test for the multiple comparison. When a non-parametric method was found applicable, Kruskal–Wallis analysis was first used and when there was a significance, the Mann–Whitney U test was used as a post hoc test.

The mRNA expression level of *psIL6* and *psIL6ns* was examined in primary spleen cells at different time points following poly I: C and ConA stimulation (Figure 5). There was no significant difference in controls (*p* = 0.5710) between different harvest time-points concerning the *psIL6* transcript level, while significant differences were observed in poly I: C-treated cells (*p* = 0.0003) and ConA-treated cells (*p* = 0.0093). At 3 h (*p* = 0.0043) and 6 h (*p* = 0.0022) post stimulation, *psIL6* transcript levels were significantly higher in the poly I: C-treated groups compared to corresponding control cells, while no significant difference was observed at 12 h (*p* > 0.9999) and 24 h (*p* = 0.0519). For ConA-treated groups at 3 h (*p* = 0.0022), 6 h (*p* = 0.0022), and 12 h (*p* = 0.0022) post stimulation, significantly higher *psIL6* transcript levels were observed compared to control cells, while no significance was observed at 24 h (*p* = 0.1143).

Similar expression pattern was observed for the *psIL6ns* mRNA expression (Figure 5b,d). There was no significant difference in controls (*p* = 0.5258) between different time-points concerning the *psIL6ns* transcript level, while significant differences were observed in poly I: C- (*p* = 0.0005) and ConA-treated cells (*p* = 0.0019). At 3 h (*p* = 0.0079) and 6 h (*p* = 0.0411) post stimulation, *psIL6* transcript levels were significantly higher in the poly I: C-treated cells than in the corresponding control cells, while no statistical significance was observed at 12 h (*p* = 0.3939) and 24 h (*p* = 0.0823). For ConA-treated groups, at 3 h (*p* = 0.0043), 6 h (*p* = 0.0022) and 12 h (*p* = 0.0260) post stimulation, significantly higher *psIL6ns* transcript levels were found compared to corresponding control cells, while no significance was observed at 24 h (*p* = 0.0823).

### 3.5. psIL6 and psIL6ns Expression Following Oral Infection with A. hydrophila

**Figure 6:**
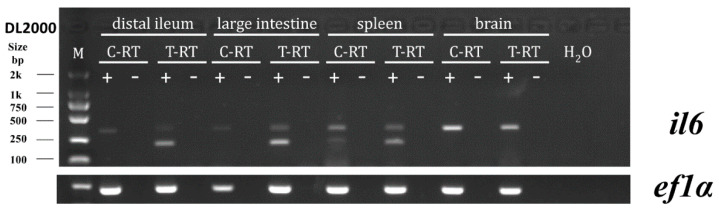
Semi-quantitative RT-PCR results of *psIL6* and *psIL6ns* expression in the brain, spleen, distal ileum, and large intestine after oral administration of *A. hydrophila. ef1α* was chosen as the reference gene. C-RT: PBS-treated turtle; T-RT: *A. hydrophila* treated turtle. + represents the addition of reverse-transcriptase when running the reverse-transcription; —represents the replacement of reverse-transcriptase with H_2_O when running the reverse-transcription. H_2_O means the template was H_2_O.

The expression of *psIL6* and *psIL6ns* mRNA in the brain, spleen, large intestine, and distal ileum was investigated at 24 h post infection from oral administration of *A. hydrophila*. Semi-quantitative RT-PCR results showed an induction of *psIL6* in the spleen, distal ileum, and large intestine of the infected group, while in the PBS-treated turtle, no *psIL6* expression was found (Figure 6). In the brain, there was no expression of *psIL6 in* either PBS-treated turtles or *A. hydrophila* infected individuals. The expression of *psIL6ns* was observed in both PBS-treated groups and *A. hydrophila* infected ones in all the tested tissues. The obtained PCR products were verified to be the target amplicons, namely *psIL6* and *psIL6ns*, by Sanger sequencing. No such PCR products were identified in negative controls, namely in samples using H_2_O as the template or in mock control (RNA), which excluded any environmental and genomic DNA contamination.

### 3.6. Spleen psIL6 Expression Following Cold Stress and Bacterial Infection

The expression of *psIL6* and *psIL6ns* mRNA in the spleen and intestines was investigated at different time points (6, 12, 24, 72 h and 7 d) following cold stress induction and bacterial infections (*S. aureus* or *A. hydrophila*).

**Figure 7:**
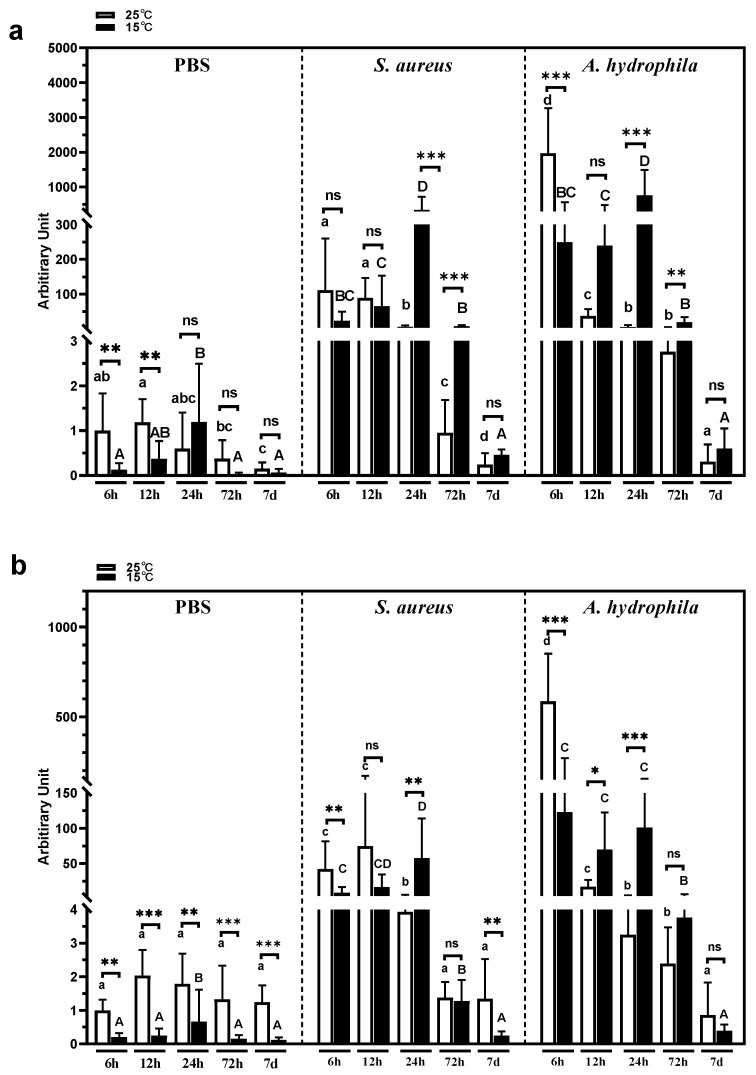
Expression of *psiIL6* (**a**) and *psIL6ns* (**b**) in the spleen after *S. aureus* and *A. hydrophila* in vivo challenge within 7 days after cold stress. The relative expression method was applied in the calculation with *ef1α* as the reference gene. The data are presented as mean + SD (*n* = 6). Capitalized and small letters: Statistical comparison between time points within a treatment group. Different letter denotes statistically significant difference (*p* < 0.05, capitalized vs. capitalized, small letters vs. small letters). (**a**) *psIL6* and (**b**) *psIL6ns* expression. * *p* < 0.05; ** *p* < 0.01; *** *p* < 0.001. ns: No statistically significant difference between the treatment groups. Two-way ANOVA analysis was carried out followed by Bonferroni’s multiple comparison test for multiple comparison. When a non-parametric method was found applicable, Kruskal–Wallis analysis was first used and when there was a significance, the Mann–Whitney U test was used as a post hoc test.

Expression of *psIL6* mRNA level in the spleen (Figure 7a) was significantly different in 25 °C-PBS groups (*p* = 0.0080) and 15 °C- PBS groups (*p* = 0.0107). In 25 °C-PBS groups, at 7 d it was significantly lower than that at 6 h (*p* = 0.0104) and 12 h (*p* = 0.0012), and at 12 h it was significantly higher than that at 72 h (*p* = 0.0059). In 15 °C-PBS groups, at 24 h it was significantly higher than that at 6 h (*p* = 0.0027), 72 h (*p* = 0.0043), and 7 d (*p* = 0.0087). Thus, we observed significant difference at 6 h (*p* = 0.0047) and 12 h (*p* = 0.0093) between the 25 °C-PBS group and 15 °C-PBS group in the spleen. When turtles were injected with *S. aureus* (Figure 7a), the expression of *psIL6* mRNA in the spleen was obviously enhanced than that in the PBS groups at 6, 12, and 24 h under normal culture temperature (25 °C), and a similar expression pattern was observed as well, in addition to 72 h, when *A. hydrophila* was used as the challenge bacteria (Figure 7a).

The highest expression of *psIL6* in the spleen appeared at 6 h and 12 h in 25 °C *S. aureus* groups and at 6 h in the 25 °C *A. hydrophila* turtles, and its expression gradually decreased during all the observed time points, which demonstrated that the strongest response under normal culture temperature appeared immediately after oral challenge with *A. hydrophila*. However, within 15 °C *S. aureus* or 15 °C -*A. hydrophila* groups, we observed the highest expression of *psIL6* mRNA appeared at 24 h, which suggested that acute cold stress led to a delayed response of *psIL6* to bacterial challenge, and significant differences were observed at 24 h (*p* = 0.0002, *S. aureus*; *p* = 0.0002, *A. hydrophila*) and 72 h (*p* = 0.0003, *S. aureus*; *p* = 0.0011, *A. hydrophila*) in either bacterial treated groups (Figure 7a).

### 3.7. Spleen psIL6ns Expression in Spleen Following Cold Stress and Bacterial Infection

Expression of *psIL6ns* in the spleen (Figure 7b) was not significantly different between the various time-points in 25 °C-PBS groups (*p* = 0.0938), while significant different *psIL6ns* expression was found in 15 °C-PBS groups (*p* = 0.0430) where it peaked at 24 h. Moreover, there were significantly different (6 h, *p* = 0.0016; 12 h, *p* = 0.0003; 24 h, *p* = 0.0070; 72 h, *p* = 0.0003; 7 d, *p* = 0.0002) *psIL6ns* expression between the 25 °C-PBS group and 15 °C-PBS at all the sampling time points. This indicated that *psIL6ns* expression was a response to the injection manipulation and cold stress. Similar to the *psIL6* expression pattern in the spleen, the *psIL6ns* mRNA level was elevated in turtles in the 25 °C-PBS group when the animals were challenged with *S. aureus* at 6, 12, and 24 h or *A. hydrophila* (Figure 7b) at 6, 12, 24, and 72 h, and the level decreased gradually. After acute cold stress, the *psIL6ns* mRNA level was increased as similarly to animals in the PBS groups at all the sampling time points. Following *S. aureus* infection (Figure 7b), the *psIL6ns* transcripts level in the spleen showed a “bell-shape” response with the peak expression at 24 h. The expression of *psIL6ns* was stable at a high level up to 24 h, and decreased beyond 72 h following *A. hydrophila* infection (Figure 7b). Significant difference was observed between the 25 °C group and 15 °C group concerning expression of *psIL6ns* in the spleen at 6 h (*p* = 0.0080), 24 h (*p* = 0.0019) and 7 d (*p* = 0..0030) following *S. aureus* challenge, and at 6 h (*p* = 0.0006), 12 h (*p* = 0.0499) and 24 h (*p* = 0.0003) after *A. hydrophila* infection (Figure 7b).

### 3.8. Intestinal psIL6 Expression Following Cold Stress and Bacterial Stimulation

**Figure 8:**
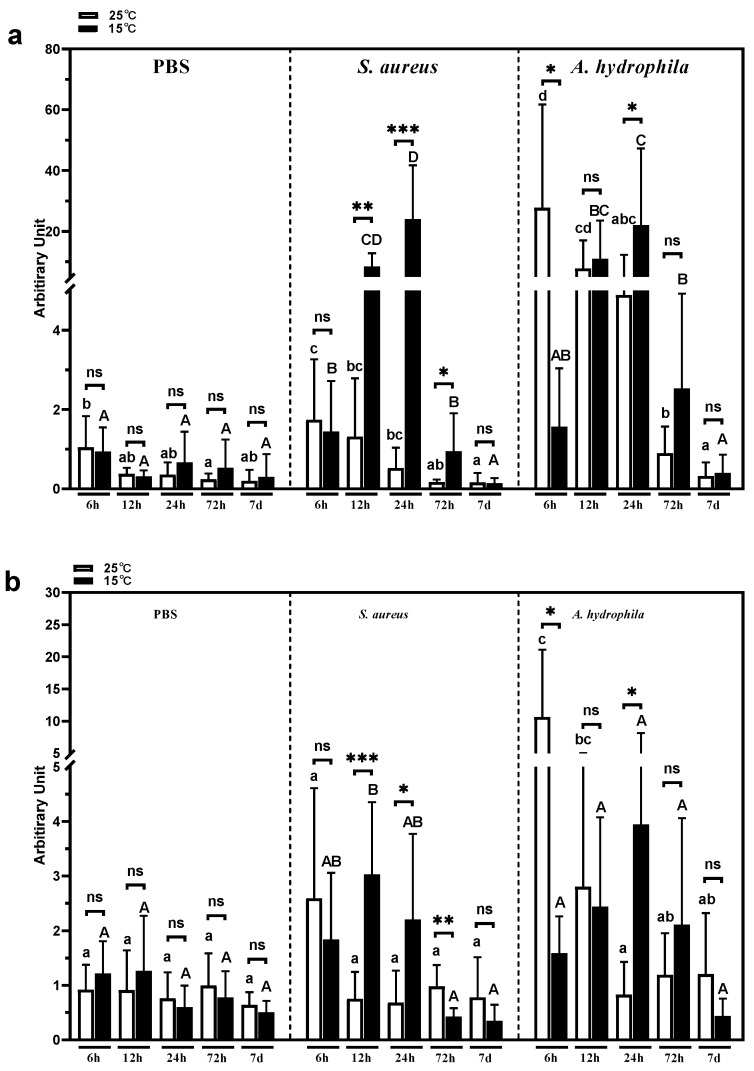
Expression of the *psIL6* and *psIL6ns* gene in the intestine (distal ileum) after *S. aureus and A. hydrophila* in vivo infection within 7 days after acute cold stress. Capitalized and small letters: Statistical comparison between time points within a treatment group. Different letter denotes statistically significant difference (*p* < 0.05, capitalized vs. capitalized, small letters vs. small letters). (**a**) *psIL6* and (**b**) *psIL6ns* expression. The data are presented as mean + SD (n = 6). * *p* < 0.05; ** *p* < 0.01; *** *p* < 0.001. ns: No statistically significant difference between the treatment groups. Two-way ANOVA analysis was carried out, followed by Bonferroni’s multiple comparison test in the multiple comparison. When a non-parametric method was found applicable, Kruskal–Wallis analysis was first used and when there was a significance, the Mann–Whitney U test was used as a post hoc test.

The expression of *psIL6* in the intestine (Figure 8a) between the various time-points was significantly different in 25 °C-PBS groups (*p* = 0.0393), with a peak at 6 h, which indicated that *psIL6ns* expression responded to PBS injection. No significant difference concerning turtle *psIL6* expression was found within 15 °C-PBS groups (*p* = 0.2552), or between the 25 °C-PBS group and 15 °C-PBS group at the same sampling time points (6 h, *p* = 0.8265; 12 h, *p* = 0.5831; 24 h, *p* = 0.4419; 72 h, *p* = 0.3904; 7 d, *p* = 0.6854). The intestinal expression of *psIL6* was at its highest at 6 h following *S. aureus* infections, and 6, 12, and 24 h following *A. hydrophila* challenge (Figure 8a). After acute cold stress, a significant higher *psIL6* mRNA level was found at 12 h (*p* = 0.0022), 24 h (*p* = 0.0007), and 72 h (*p* = 0.0121) in *S. aureus* challenged group (Figure 8a), and at 24 h (*p* = 0.0289) in *A. hydrophila* infected group (Figure 8a)—in comparison to the 25 °C group. This indicated a delayed response of *psIL6* to bacterial challenge after acute cold stress.

### 3.9. Intestinal psIL6ns Expression Following Cold Stress and Bacterial Stimulation

In the intestine, the expression pattern of *psIL6ns* in the 25 °C-PBS group was similar to the splenic *psIL6ns* expression. No significant difference between the various time-points was observed (*p* = 0.6716) (Figure 8b). No statistically significant differences of *psIL6ns* expression were observed in 25 °C-PBS groups (*p* = 0.6756) or 15 °C-PBS groups (*p* = 0.1047). *psIL6ns* expression was not statistically significantly different between 25 °C-PBS turtles and 15 °C-PBS ones at the same sampling time points (6 h, *p* = 0.3552; 12 h, *p* = 0.4378; 24 h, *p* = 0.5129; 72 h, *p* = 0.4468; 7 d, *p* = 0.2849). The expression of *psIL6ns* in the intestine was increased at 6 h following both *S. aureus* and *A. hydrophila* infections (Figure 8b). After acute cold stress, compared to its corresponding 25 °C infected groups at the same sampling time points, a significant higher *psIL6ns* mRNA level was found at 12 h (*p* = 0.0093) and 24 h (*p* = 0.0140) in the *S. aureus* challenged group (Figure 8b), and at 24 h (*p* = 0.0027) in the *A. hydrophila* infected group (Figure 8b). This may imply a delayed response of *psIL6ns* to bacterial challenge after acute cold stress.

## 4. Discussion

In the present work, we identified two complete cDNA sequences of *IL6* by RACE, namely *psIL6* and *psIL6ns* with different lengths in the Chinese soft-shelled turtle. The semi-quantitative RT-PCR analysis showed that the Chinese soft-shelled turtle possessed variants of *IL6*—appearing as two clear bands with the expected molecular weights. These two PCR products were sequenced by Sanger sequencing and were confirmed to be two variants.

The following analysis by reciprocal BLAST in GenBank and multiple alignments of the deduced amino acid sequences suggested that *psIL6* and *psIL6ns* were homologous to mammalian IL6 (Figure not shown). Similar to other IL6 homologs [3,5,9,13], a typical IL6/G-CSF/MGF family displaying signature certain characteristics (C-X(9)-C-X(6)-G-L-X(2)-Y/F-X(3)-L) was found in the deduced *psIL6* and *psIL6ns*. Moreover, with online software SMART, an IL6 domain was also found in the deduced proteins. This suggested that the two molecules were mammalian *il6* counterparts. This suggestion was further strengthened by phylogenetic analysis. In the phylum, the two IL6s only locate in the IL6 clades, not in any other IL6 family clades. It was found that turtle IL6s formed a subunit, which grouped with the avian IL6 subunit. The bootstrap values were not very high—which weakened the suggestion that the turtle IL6s and avian formed a subunit. However, this is not unexpected since cytokines such as IL6 evolve rapidly like other cytokines in the immune system [27,28]. Based on the bioinformatic analysis, we concluded that *psIL6* and *psIL6ns* were homologous to mammalian *il6*.

The comparison of *psIL6* and *psIL6ns* cDNA sequences further confirmed the occurrence of two *psIL6s*, where one contained 125bp additional bases. This implied that the two cDNA were more likely splice variants rather than transcripts from two different paralogs. Several other findings gave support to our conclusion: (1) At present, no duplicated IL6 genes have ever been found in vertebrates ranging from teleost to mammals [2,3,4,5,6,7,8,9,10,11,12,14]; (2) Duplicated genes are less likely to share the same 3′ UTRs [29]; (3) when *psIL6* and *psIL6ns* cDNA sequences were BLASTed against the genomic sequence, they hit the same gene which was denoted as *IL6*—while no other genes or loci matched in similar fashion.

Moreover, gene synteny analysis also suggested that the turtle *IL6* was flanked by *sp4*, *cdca7l*, and *tomm7* genes, which have been reported to be very conserved during evolution (Supplementary Figure S3) [9]. In addition, the results from BLAST demonstrated that *psIL6* is corresponding to a 5-exon-4-intron structure in genomic DNA, whereas *psIL6ns* kept the first intron during splicing, which corresponds to a 4-exon-3-intron structure. Keeping the first intron in the *psIL6ns* introduces a frameshift which leads to a premature stop. An alternative translation starting site (ATG) was found downstream of the stop codons, which is in the same frame of *psIL6* translation and yields a truncated protein lacking 16 amino acids at the N-terminal end. No deduced signal peptide was found in the truncated protein by Signal P4.1 analysis, which was not surprising as the predicted signal peptide is the initial 28 amino acids encoded by the short splicer. Therefore, we named the longest splicer *psIL6ns*, and the shorter one *psIL6*.

Different splicers of *IL6* have been observed in mammals, such as human [30,31,32,33,34], mouse [35], and wallaby (*Macropus eugenii*) [36]. In human tissues and tumor cell lines, alternative spliced deletion has been observed for exon 2 and/or exon 4 [30,31,32,33,34]. In mice placenta and in stimulated spleen, both whole and partial exon deletion during RNA splicing have been identified [35]. In wallaby, an alternative splicing variant lacking whole exon 2 was found [36]. In these animals, the loss of exon 2 resulted in a lack of signal peptide. In the current study, although *psIL6ns* does not lose any exon, the encoded protein did not have signal peptide either, which is due to the maintenance of intron 1 during splicing. This is a new mechanism to generate an IL6 isoform without signal peptide. The results herein suggest alternative splicing of *IL6* RNA, and expands knowledge relevant to non-mammalian species. Splicing isoforms of other genes have been found in Chinese soft-shelled turtles [37,38], the precise physiological significance of alternative splicing is not known.

In human IL6, the residues F201, R207, and R210 at the C-terminal end play a pivotal role in receptor binding and bioactivity [39], and they are much conserved non-fish vertebrates as shown in this study (Figure 2). In human IL6, two disulfide bonds, cys72-cys78 and cys101-cys111, have been found, and they both play roles in IL6’s bioactivity, although the latter is functionally more important [40]. However, only the latter disulfide bond was found in the selected fish IL6s [5,6,7,8,9,10,11,12]. In the Chinese soft-shelled turtle, the four cysteines were conserved which suggests that turtle IL6 possesses a similar function as its mammalian counterparts.

Constitutive expression of *psIL6* and *psIL6ns* was found in several tissues, namely liver, spleen, kidney, lung, stomach, distal ileum, and large intestine with qRT-PCR detection. This is in line with previous findings made from studies on several vertebrates [10,11,12,41]. However, when semi-quantitative RT-PCR was used in the current study, *psIL6* was found to be constitutively expressed only in the spleen, while *psIL6ns* was constitutively expressed in all the sampled tissues (brain, spleen, distal ileum, and large intestine). This is consistent with several observations that only limited number of tissues were found to constitutively express *IL6* mRNA in other animal species [5,6,7,8], where also semi-quantitative RT-PCR was used. It is well known that qRT-PCR is much more sensitive than semi-quantitative RT-PCR, and different sensitivity of the two methods is likely an attribution to the differences seen. A high expression of *psIL6* was observed in the spleen which may suggest it plays a role in immune response as its counterparts in other vertebrates do [2,10,13]. In the yellow croaker, poly I: C-injection fish showed a higher level of *IL6* expression in the spleen, head kidney, and liver [11], which is consistent with our observation in the Chinese soft-shelled turtle. In primary spleen cells, a significantly up-regulated expression of *psIL6* was observed after poly I: C treatment at 6 h post stimulation. Moreover, the level of *psIL6* mRNA was significantly enhanced after intraperitoneally bacterial challenge in the spleen and distal ileum. In addition, at 24 h after oral administration with *A. hydrophila*, *psIL6* was greatly induced in the spleen, distal ileum, and large intestine. Therefore, it is concluded that *psIL6* functions in the Chinese soft-shelled turtle immune response.

To our surprise, *psIL6ns* was higher expressed in all the selected tissues compared to *psIL6*, and also in response to poly I: C and ConA in spleen cells and after bacterial infection. As the *psIL6ns* encoded protein was predicted to contain no signal peptide, *psIL6ns* is not supposed to be secreted from cells. Prokaryotic recombinant human IL6 with lack of any signal peptide has been found to possess biologic activity which is relatively tissue specific, with high activity on myeloid cells and relatively minor activity on B-cells/plasma cells [31]. However, as produced by a bocavirus expression system, human IL6 isoform without exon 2 was found to be a competitive inhibitor to the full length IL6 [33]. It has been argued that the missing of codons in helix A, which is also located in exon 2, rather than the deletion of signal peptide in exon 2 may contribute to the differences in the bioactivities. This is because Helix A is involved in the IL6/IL6R complex binding to gp130 [32]. This explanation was further strengthened by the observation that the deletion of first 28 amino N-terminal residues (preceding helix A) had no effects on human IL6 bioactivities [42]. *PsIL6ns* and *psIL6* possess only differences with regards to the signal peptide, and their 3D structures (Supplementary Figure S4) exactly match each other. It may be less likely that *psIL6ns* functions as an agonist to *psIL6* since it is not secreted to become an extracellular cytokine.

IL6 plays important roles in immune response, acute-phase reactions, hematopoiesis, and inflammations in various tissues [43]. Its expression could be induced by many stressors including psychological stress, physiological excises, and acute environmental stress, which have been observed in mammals and fish [12,44,45,46,47]. In rat liver, lung, and brain, the IL6 level has been described to be significantly up-regulated upon cold stress [48]. In mice peritoneal macrophages, the LPS (bacterial lipopolysaccharide)-induced IL6 secretion was observed to be augmented by cold water/medium stress [49]. In the chicken, IL6 was found to be regulated by a heat shock factor, which is a stress response molecule [50], and the *IL6* level was significantly enhanced in peripheral blood leucocytes by cold stress [47]. These are cases of IL6 in endothermic animals where its expression could be affected by cold stress. In ectothermic taxa, there is to our knowledge, no previous report on IL6 induction by cold stress except findings in the Chinese soft-shelled turtle reported in our previous paper [16]. Quite recently, the transcription of *IL6* in the blunt snout bream and pufferfish (*Takifugu obscurus*) was found to be enhanced by ammonia stress [12,51], which indicated that also fish IL6 possesses function in stress response. The Chinese soft-shelled turtle also faces environmental stress during its culture, and is very sensitive to environmental temperature fluctuation [16,52]. Farmers frequently reported mass mortalities of the Chinese soft-shelled turtle during the period of acute temperature variation [16]. Thus, in this study, we further analyzed *IL6* mRNA expression of the Chinese soft-shelled turtle under acute cold stress and/or bacterial challenge in the spleen and intestine, which are import for turtles fighting infection. In our previous study [16], it has been found that the expression of the Chinese soft-shelled turtle *IL6* in the acute cold-stressed group was higher than in the controls at 24 h after *A. hydrophila* challenge. However, it is not valid to conclude that *IL6* expression in response to bacteria was enhanced by acute cold stress, as temporarily changes of cytokines expression could was not recorded [53]. Therefore, in the current experiment, five sampling time points (6, 12, 24, 72 h and 7 d post infection) were selected to obtain an expression profile over time. The current study identified that both *psIL6* and *psIL6ns* expression were regulated during bacterial infection, and the peak production was postponed by acute cold stress in the spleen and intestine. As an ectothermic animal, the physiological and biochemical processes of the Chinese soft-shelled turtle is controlled by the environmental temperature, and low temperatures below the optimal temperature range normally decrease biochemical processes. Therefore, this delay may likely be attributed to the low temperature. Whether this delay may cause severe immune impairment of the Chinese soft-shelled turtle in response to the infection is important to be addressed in future studies.

In conclusion, the present study has identified for the first time that turtles possess a functional *IL6* counterpart with signal peptide (*psIL6*). Interestingly, *psIL6ns* and *psIL6* both were slightly differentially regulated during immunostimulation in vitro, during cold stress and bacterial challenge in vivo, which implied that *psIL6ns* may represent a functional isoform as well.

## Supplementary Materials

The following are available online at https://www.mdpi.com/2079-7737/9/5/111/s1, Figure S1: Amplicons of *psIL6* and *psIL6ns* sequences spanning ORFs in the verification PCR amplification, Figure S2: Genomic structure of *IL6s* and the relationship *psIL6* and *psIL6ns* mRNA to genomic DNA. (**A**) Genomic structure of *IL6* in several selected vertebrates; (**B**) the relationship *psIL6* and *psIL6ns* mRNA to genomic DNA, Figure S3: Gene synteny of *IL6s* in selected vertebrates, Figure S4: The predicted 3D structure of *psIL6* and *psIL6ns*. (**A**) *psIL6* and (**B**) *psIL6ns* 3D structure were predicted SWISS-MODEL based on homology-modelling method, Figure S5: The primers for *psIL6* and *psIL6ns* amplification with qRT-PCR were demonstrated. Table S1: The primers used in this study, Table S2: Intron-exon junctions and flanking nucleotides of *IL6* gene of Chinese soft-shelled turtle, Table S3: Similarity and positives comparison of *IL6* between *Pelodiscus sinensis* and other species, Table S4: Accession number of selected *IL6* proteins from different species in GenBank or ENSEMBL.
