# Supplementary material for: A New IL6 Isoform in Chinese Soft-Shelled Turtle (Pelodiscus sinesis) Discovered: Its Regulation during Cold Stress and Infection
Source: Biology (Basel). 2020 May 25;9(5):111. doi: 10.3390/biology9050111 (PMC7284502; doi:10.3390/biology9050111)
Supplement: Supplementary file 1 [file biology-09-00111-s001.pdf]

Supplementary Figure S1.

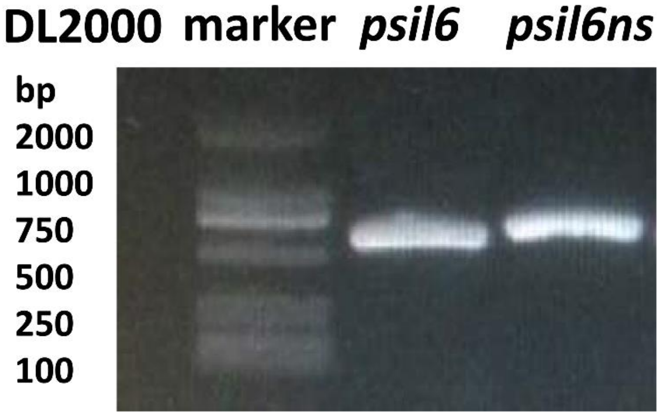

Supplemental Figure S1 Amplicons of *psIL6* and *psIL6ns* sequences spanning ORFs in the verification PCR amplification.

Supplementary Figure S2.

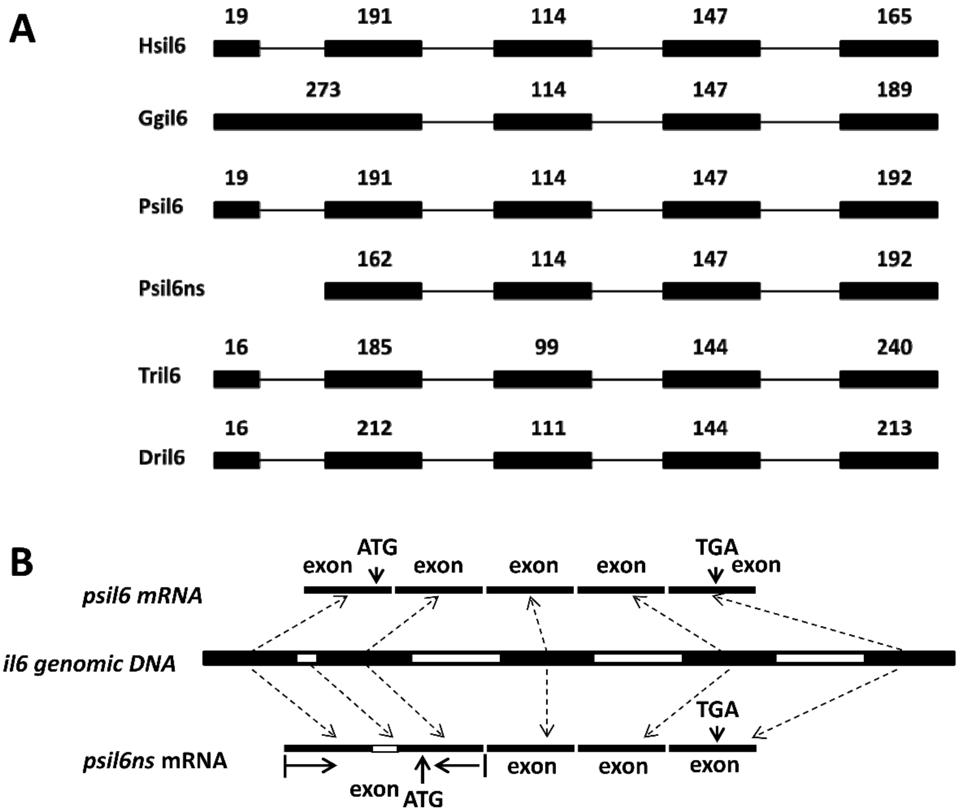

Supplementary Figure S2 Genomic structure of *IL6*s and the relationship *psIL6* and *psIL6ns* mRNA to genomic DNA. (A) Genomic structure of *IL6* in several selected vertebrates; (B) the relationship *psIL6* and *psIL6ns* mRNA to genomic DNA.

Supplementary Figure S3

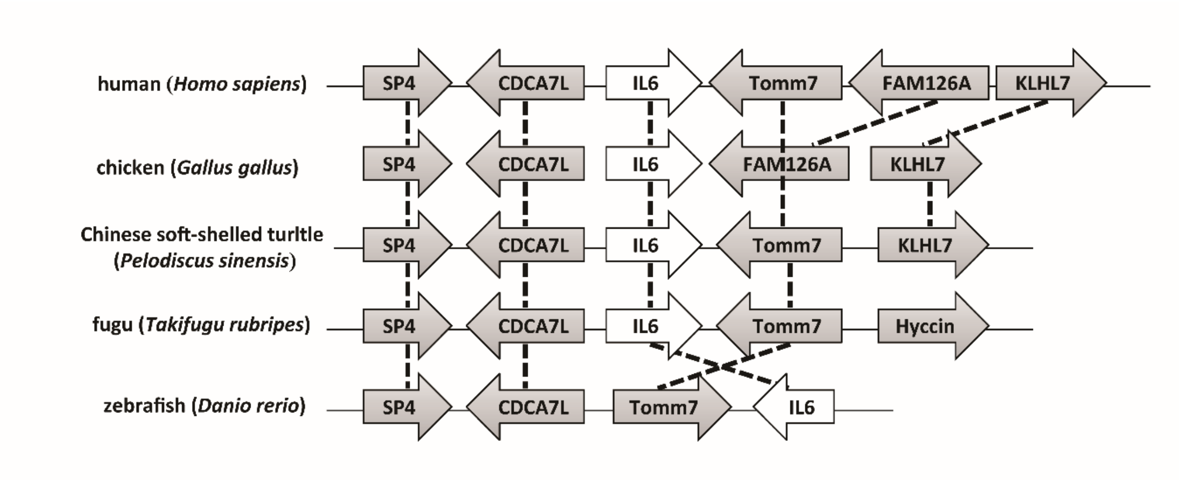

Supplemental Figure S3 Gene synteny of IL6s in selected vertebrates.

Supplementary Figure S4

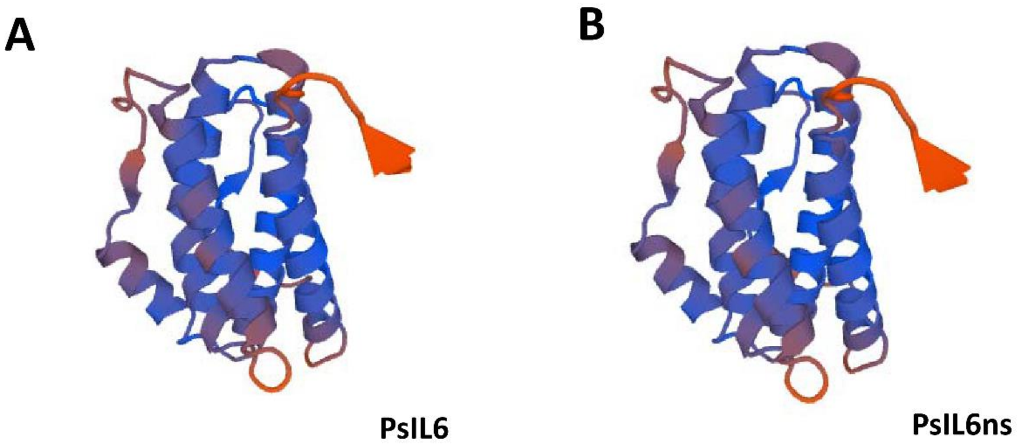

Supplemental Figure S4 The predicted 3D structure of psIL6 and psIL6ns. (A)psIL6 and (B) psIL6ns 3D structure were predicted SWISS-MODEL based on homology-modelling method.

Supplementary Figure S5

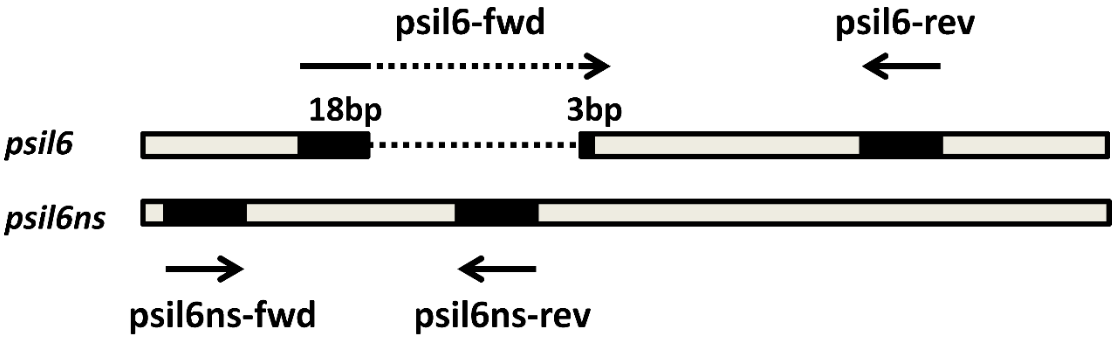

Supplementary Figure S5 The primers for *psIL6* and *psIL6ns* amplification with qRT-PCR were demonstrated.

Supplementary Table S1 The primers used in this study.

Primer name	Sequence（5’→3’）	Application
psIL6-fwd1	CTCCCCAAGATCACAGAGGA	3’-RACE
psIL6-fwd2	CTCATCTCATCCTCCGGGACTT	3’-RACE
psIL6-fwd3	TCTCCCACTGTCCTTTTGAGGGA	3’-RACE
psIL6-fwd4	TGGGTAGCAACAGCCCTCACA	3’-RACE
psIL6-rev1	TAGCCATGCCAAGGACTCGCAAT	5’-RACE
psIL6-rev2	GGGGAGGTTCAGATCATTTTGGGCA	5’-RACE
psIL6-verfwd	AGACCTCTGGCCAGCAACTCTT	Verification
psIL6-verrev	ACTACACTGTCATCTGCCACATGA	Verification
psIL6-fwd	GAATTATCTTCGGGGATGCTC	qRT-PCR
psIL6-rev	TCGTCCTTCAGTTTAGCAGCTT	qRT-PCR
psIL6ns-fwd	TCTGGCCAGCAACTCTTTAGA	qRT-PCR & semi-quantitive RT-PCR
psIL6ns-rev	AGACAAGGAAGGAGTGGAATGAA	qRT-PCR
psIL6-rtrev	ATGCCAAGGACTCGCAATCT	semi- quantitive RT-PCR
psef1α-fwd	CCACCAACTCGTCCAACTGA	qRT-PCR
psef1α-rev	ACCAACATTGTCACCAGGCA	qRT-PCR

Supplementary Table S2 Intron-exon junctions and flanking nucleotides of *IL6* gene of Chinese soft-shelled turtle.

Intron	Sequence	Intron size
IL6-1	GAT gt⋯ag GCT	125bp
IL6-2 (IL6ns-1)	GAG gt⋯ag ATG	717bp
IL6-3(IL6ns-2)	GAG gc⋯ag GAG	174bp
IL6-4(IL6ns-3)	ATG gt⋯ag GTG	2264bp

Supplementary Table S3 Similarity and positives comparison of IL6 between *Pelodiscus sinensis* and other species.

Species	Accession number	Identities (%) IL6/IL6ns	Positives (%) IL6/IL6ns
Homo sapiens	NP_000591.1	39	62
Mus musculus	NP_112445.1	35	57
Gallus gallus	ADL14564.1	68	80
Chrysemys picta bellii	XP_008169102.1	90	95

Oncorhynchus mykiss	NP_001118129.1	29	46
Danio rerio	NP_001248378.1	26/27	42/43
Salmo salar	CEH11582.1	24	40
Takifugu rubripes	NP_001027894.1	23	37

Supplementary Table S4 Accession number of selected IL6 proteins from different species in GenBank or ENSEMBL.

Common name	Species name	Acc. no
Human	*Homo sapiens*	NM_013246
Mouse	*Mus musculus*	NM_019952
Cat	*Felis catus*	XM_023240108
Chicken	*Gallus gallus*	XM_015272641
Anolis	*Anolis carolinensis*	XM_016994570
Chinese alligator	*Alligator sinensis*	XM_025216129
Crocodylus	*Crocodylus porosus*	XM_019556642
Painted turtle	*Chrysemys picta bellii*	XM_008178280
Chinese soft-shelled turtle	*Pelodiscus sinensis*	XM_006112962
Green sea turtle	*Chelonia mydas*	XM_007053786
Mexican Box Turtle	*Terrapene mexicana triunguis*	XM_024220025
Lizard	*Pogona vitticeps*	XM_020806024
Frog	*Xenopus laevis*	XM_018225579
Zebrafish	*Danio rerio*	XM_685641
Fugu	*Takifugu rubripes*	XM_011610642
Rainbow trout	*Oncorhynchus mykiss*	XM_021582994
Croaker	*Larimichthys crocea*	XM_010735557
Flounder	*Paralichthys olivaceus*	XM_020083323
